# Antinociceptive action of *Vanillosmopsis arborea* in male mice

**Published:** 2017

**Authors:** Laura Inocencio Leite, Gerlânia de Oliveira Leite, Bruno Anderson Fernandes Silva, Severino Denício Gonçalves Sousa, Thales Silva Coutinho, Renata Souza Sampaio, Irwin Menezes, José Galberto Martins Costa, Adriana Rolim Campos

**Affiliations:** 1 *Department of Biology at the Federal University of Cariri, Brejo Santo – CE, Brazil*; 2 *Department of Pharmacology, University of Santa Maria, Santa Maria- RS, Brazil*; 3 *Department of Chemical Biological at the Regional University of Cariri, Crato – CE, Brazil *; 4 *Department of Pharmacology at the University of Fortaleza, Fortaleza – CE, Brazil*

**Keywords:** Vanillosmopsisarborea, Essential oil, Antinociceptive, Antipruritic

## Abstract

**Objective:**

*Vanillosmopsis arborea* Baker (Asteraceae) has high economic value from Chapada to Araripe and its bark essential oil is a potential source of alpha-bisabolol. The present study aimed to elucidate the antinociceptive and antipruritic properties of the essential oil of *V. arborea *Baker (EOVA) in mice.

**Materials and Methods:**

The antinociceptive activity was assessed using the capsaicin, glutamate, hot plate and cold allodynia tests. The antipuritic effects were also verified based on histamine-induced scratching behavior.

**Results:**

EOVA reduced the paw licking induced by capsaicin, but not that induced by glutamate. The essential oil increased the latency time in the hot plate, attenuated the cold allodynia induced by acetone and inhibited histamine-induced scratching behavior.

**Conclusion:**

The experimental data demonstrated that EOVA showed central and peripheral antinociceptive activity and antipruritic effect.

## Introduction

Pain, an unpleasant sensory and emotional experience associated with actual or potential tissue damage (IASP, 1994 ▶), affects approximately 70% of world population and is associated with deficit in the quality of life (WHO, 2007 ▶). 

Mechanical pinprick hyperalgesia is a very distressing symptom presented by approximately 29% of patients with neuropathic pain. During, a state of hyperalgesia, an already nociceptive stimulus is perceived even more painful (Baron et al, 2010 ▶). Secondary hyperalgesia develops in uninjured area surrounding a damaged nerve, by modulation of the spinal and supraspinal nociceptive system (Simone et al, 1991 ▶; Westlund, 2006 ▶). It can be induced experimentally in healthy humans by the heat/capsaicin model (Petersen and Rowbotham, 1999 ▶).

Glutamate is one of the important excitatory neurotransmitters that participates in many physiological and pathological states as nociception and hypersensitivity (Dickenson, 1997 ▶; Coderre, 1993 ▶) central nervous system injury (Lipton and Rosenberg, 1994 ▶; Hudspith, 1997 ▶). Although there is evidence indicating that glutamate has a relevant role in the process of pain transmission, it has not been completely established, however, whether glutamate is able to directly produce nociception when it is injected into the rat paw (Teixeira, 2001 ▶). 

*V. arborea *Baker is native to the Araripe National Forest, in the Northeast of Brazil in the state of Ceará. There are few studies concerning the traditional use of this plant. However, some biological and pharmacological studies have shown that its essential oil presents antinociceptive activities (Santos et al., 2015 ▶; Leite et al, 2014 ▶).

This study aimed to evaluate the central and peripheral antinociceptive activity as well the antipruritic effect of the essential oil from *V. arborea*.

## Materials and Methods

**Obtaining the essential oil**

The essential oil from *V. arborea* bark (EOVA) was obtained in the Natural Products Research Laboratory of Regional University of Cariri. Phytochemical analysis revealed the presence of (-)-α-bisabolol (97.9%), methyl eugenol (1.6%) and bisabolol oxide (0.5%).

**Animals**

Male Swiss albino mice (20-25 g; n=8) obtained from the Central Animal House of Regional University of Cariri, were housed in environmentally controlled conditions (22º C,; 12 hr-12 hr light-dark cycle), with free access to standard pellet diet (Purina, São Paulo, Brazil) and water. Animals were kept in cages with raised floors to prevent coprophagy. All experimental protocols were in accordance with the ethical guidelines of National Council for the Control of Animal Experimentation, Brazil.

**Capsaicin test**

Capsaicin was used to induce nociception as previously described by Gadotti et al (2005) ▶. Groups were treated with vehicle (2% Tween 80, 10 ml/kg, p.o.), EOVA (25, 50, 100 and 200 mg/kg, p.o.) or morphine (7.5 mg/kg, s.c.). Then 30 or 60 min after the treatments, animals received capsaicin (1.6 μg in 20 μl, intraplantar). The time spent for licking the injected paw in seconds (sec was recorded for a period of 360 sec. 

**Glutamate test**

Glutamate was used to induce nociception as previously described by Beirith et al (2002) ▶. Treatments were the same as those in capsaicin test. Then, 30 or 60 min after the treatments, animals received capsaicin (20 mmol/paw, intraplantar). The time spent for licking the injected paw in sec was recorded for a period of 900 sec. 

**Hot plate test**

The hot-plate test (Eddy et al, 1953 ▶) was used to evaluate the central antinociceptive effect of EOVA. Here, the time latency to lick either hind paw or to jump up (reaction time) when placed on a hot-plate (55.0 ± 1.0 ºC) (Ugo Basile, model-DS 37, Italy), was recorded. Mice showing a pretreatment reaction time greater than 15 sec in the hot-plate test were not used in the experiment. A cut off time of 45 sec was used to avoid tissue damage. Treatments were the same as those in capsaicin and glutamate tests. The reaction time was recorded before (0) and 0.5, 1, 1.5, 24 and 48 hr after the treatments. 

**Cold sensitivity**

To assess cold allodynia (Choi et al, 2012 ▶) a drop (50 µl) of acetone was placed against the centre of the plantar surface of the hind paw and a stopwatch was started. Groups were treated with vehicle (2% Tween 80, 10 ml/kg, p.o.), EOVA (25, 50, 100 and 200 mg/kg, p.o.) or morphine (7.5 mg/kg, s.c.). Mice response was monitored in the first 20 sec after acetone application. If the mouse did not withdraw, flick or stamp its hind paw within this 20 sec period, then no response was recorded for that trial (0). However, if within this 20-sec period the animal responded to the cooling effect of the acetone, then the animal's response was assessed for an additional 20 sec (a total of 40 sec from initial application). Responses to acetone were graded according to the following four-point scale: 0, no response; 1, quick withdrawal, flick or stamp of the paw; 2, prolonged withdrawal or repeated flicking (more than 2 times) of the paw; 3, repeated flicking of the paw with licking directed at the plantar surface of the hind paw. Acetone was applied alternately three times to each hind paw and the responses were scored categorically. Cumulative scores were then generated for each mouse. 

**Histamine-induced scratching behavior**

Groups were treated with vehicle (2% Tween 80, 10 ml/kg, p.o.), EOVA (25, 50, 100 and 200 mg/kg, p.o.) or dexchlorpheniramine maleate (2 mg/kg, p.o.). Then, 60 min after the treatments, histamine (100 nmol) was injected into the neck intradermally (applied 20 µl). After histamine injection, bouts of scratching were counted for 1 hr.

**Statistical analysis**

The results are presented as mean±SEM. for 8 animals per group. Statistical comparisons of the data were made by one-way analysis of variance (ANOVA), followed by Newman Keuls test for multiple comparisons using Graph Pad Prism, version 4.0. Differences were considered statistically significant at p< 0.05.

## Results

**Capsaicin, glutamate and hot-plate tests**

Table 1 shows that EOVA significantly diminished the paw-licking response to subplantar injection of capsaicin when compared to vehicle-treated group (control). EOVA at all doses was not able to inhibit glutamate-induced licking time (Table 1). EOVA 50 (0.5 hr) and 200 mg/kg (1 and 24 hr) groups manifested significant antinociception as compared to control group (Table 2).

**Table 1:** Effect of EOVA on capsaicin and glutamate tests in mice

**Groups**	**Dose** **(mg/kg)**	**Paw licking time (sec)**	
		**Capsaicin**	**Glutamate**
**Control**	-	45.17±5.250	43.83±6.498
**EOVA**	25	22.75±2.401****	48.00±11.81
	50	15.25±0.881****	30.67±6.427
	100	6.375±1.438****	53.33±13.60
	200	21.38±3.289****	52.00±9.688
**Morphine**	7.5	3.875±2.401****	19.56±4.198**

Data are presented as mean ± SEM of 8 mice/group.

** p˂ 0.01; and

**** p ˂ 0.0001 vs control (ANOVA, Newman Keuls test).

**Cold sensitivity and histamine-induced scratching behavior**

Administration of EOVA (25, 50, 100 and 200 mg/kg, p.o.) attenuated the cold allodynia induced by acetone. EOVA at 25 and 200 mg/kg significantly inhibited histamine-induced scratching behavior in mice (Table 3). 

**Table 2:** Effect of EOVA on hot-plate test

**Groups**	**Dose** **(mg/kg)**	**Reaction time (sec)**					
		**0hr**	**0.5hr**	**1hr**	**1.5hr**	**24hr**	**48hr**
**Control**	-	14.96±1.21	13.14±1.25	13.53±1.67	14.46±0.72	13.15±0.63	15.03±1.11
**EOVA**	25	10.68±0.45	17.75±1.17	13.21±0.89	14.46±1.05	10.15±0.51	10.65±0.95
	50	16.90±2.38	19.56±1.94**	14.31±0.69	13.53±0.86	14.46±1.30	11.31±0.58
	100	14.62±1.63	15.62±1.42	14.96±0.86	17.93±1.52	12.84±0.73	11.25±0.75
	200	9.65±0.73	14.87±1.52	20.50±1.31**	14.84±1.12	18.73±1.39*	11.50±0.55
**Morphine**	7.5	12.62±1.72***	24.50±1.50***	27.62±1.45**	24.62±1.34**	25.50±1.99	22.62±1.46*

Values represent mean ± SEM. p < 0.05;

** p < 0.01; and

*** p<0.001 as compared to the control group (ANOVA, Student-Newman Keuls).

**Table 3:** Effect of EOVA on acetone-induced cold sensitivity and histamine-induced scratching behavior

**Groups**	**Dose (mg/kg)**	**Cold sensitivity**	**Scratching behavior**
		**Reaction time (sec)**	**Number of scratches (60 min)**
**Control**	-	91.00±4.21	45.17±5.25
**EOVA**	25	52.00±5.83	56.00±8.03
	50	75.17±3.37	87.17±16.02
	100	42.00±6.32***	90.00±21.14
	200	49.67±4.75***	43.50±11,15**
**Morphine**	7.5	34.29±4.62***	-
**Dexchlorpheniramine maleate **	2	-	29.33±1.66****

Values represent mean ± SEM.

** p< 0.01;

*** p< 0.001

**** p ˂ 0.0001 as compared to the control group (ANOVA, Student-Newman-Keuls).

## Discussion

Pain control is one of the most important therapeutic priorities. In normal conditions, this activity is associated with and afferent impulses (C and A) of the peripheral nerves that are activated by various stimuli (thermal, mechanical or chemical). Several pathological diseases and tissue injuries result in the pain. This response results in local release of a variety of chemicals that act on nerve endings, either by activating them directly, or increasing their sensitivity to other form of stimulation (Rang et al, 2004 ▶).

Leite et al., 2014 ▶) previously demonstrated the antinociceptive activity of essential oil *Vanillosmopsis arborea* (EOVA) on topical corneal pain. Thus, this research attempted to provide a better understanding of the therapeutic utility of this oil and its mechanisms of action. It should be also taken into account that the oil is a mixture of compounds; however, by this activity may be related to its major component, (-)-α-bisabolol.

EOVA, at all doses, significantly reduced capsaicin-induced nociceptive behaviors. These results are in contrast with our previous study which reported that EOVA was not able to decrease the capsaicin-induced ear edema (Leite et al, 2011 ▶). This difference between results can be justified by minor absorption of EOVA via transdermal route, a fact observed in other studies (Wokvich et al., 2006 ▶). Hypothesis reinforced since the EOVA was effective in preventing visceral nociception induced by capsaicin (Benson, 2005 ▶).

EOVA could not revert the nociceptive behavior induced by glutamate, suggesting that there is no involvement of glutamatergic neurons in the EOVA antinociceptive effects. 

The antinocicepitve effect of EOVA on hot-plate test suggests a supraspinal activity, as it is known that this test is sensitive to opioids and other substances that act on the central nervous system.

We decided to investigate the antiallodynic effect of EOVA against topical acetone. This stimulus is not harmful, and activates the free nerve endings sensitive to cold. EOVA, at all doses, increased cold nociceptive threshold and this can be related to a sensitization of supraliminal stimuli. Mediators involved in this test have an important role in the development of nociceptive response induced by different stimuli (Cunha et al, 2008 ▶).

It was first shown, in our study the antipruriginous effect of EOVA. According to Dogrul et al. (2001) ▶, the itch caused by histamine presents a nociceptive component. So, we believe that EOVA reduces the pruritus by modulation of the pain process. This EOVA effect may be related to the ability of this oil to modulates pain by affecting the serotoninergic neurotransmission (Leite et al., 2014 ▶).

These results demonstrate antipruritic and antinociceptive potentials of EOVA, suggesting a central mechanism exerting downward inhibition. 
